# Urine fibroblast growth factor 23 levels in hypertensive children and adolescents

**DOI:** 10.3325/cmj.2015.56.344

**Published:** 2015-08

**Authors:** Magdalena Zając, Agnieszka Rybi-Szumińska, Anna Wasilewska

**Affiliations:** Department of Pediatrics and Nephrology, Medical University of Białystok, Białystok, Poland

## Abstract

**Aim:**

To determine the correlation of urinary fibroblast growth factor 23 (FGF23) excretion with blood pressure and calcium-phosphorus metabolism.

**Methods:**

The study included 42 hypertensive (17 girls) and 46 healthy children and adolescents (17 girls) aged 6-18 years admitted to the Department of Pediatrics and Nephrology, Medical University of Białystok between January 2013 and December 2013. FGF23 in urine was measured using Human Intact FGF-23 ELISA Kit.

**Results:**

Hypertensive participants had significantly higher urine FGF23/creatinine values than the reference group (8.65 vs 5.59 RU/mg creatinine, *P* = 0.007). Urine FGF23/creatinine positively correlated with systolic blood pressure in all participants. In hypertensive patients, urine FGF23/creatinine positively correlated with serum calcium and negatively with serum 25(OH)D, urinary calcium, phosphorus, and magnesium.

**Conclusion:**

This study found that FGF23 may play an important role in the pathogenesis of hypertension in children and adolescents, but our results should be confirmed by further studies.

Hypertension is a chronic medical condition and a major risk factor for cardiovascular disease, heart failure, and chronic kidney disease (CKD). Hypertension was found to be associated with several factors, among them calcium-phosphorus imbalance, lack of vitamin D, and serum parathyroid hormone (PTH) (1-5). However, far too little attention has been paid to phosphates and hormonal mechanisms responsible for their regulation, especially since the consumption of phosphorus has considerably increased in recent years. Some studies have shown that serum phosphorus increases BP (11,12). However, recent studies have found that high phosphorus intake reduces BP, when the diet is rich in calcium (6-8), while other have shown that BP was reduced by low phosphorus and high calcium diet (9,10).

Phosphate concentration is primarily regulated by PTH and fibroblast growth factor 23 (FGF 23) – phosphatonin, produced by osteoblasts/osteocytes in the bone, which, similarly to PTH, stimulates phosphaturia. FGF23 decreases renal calcitriol production and inhibits PTH secretion. Its main function is to maintain phosphate homeostasis by increasing urinary phosphate excretion and decreasing serum 1,25(OH)_2_D (13,14) In patients with CKD, it positively correlated with PTH secretion (15,16). The increase in FGF23 in those patients led to an early development of secondary hypertension by suppression of 1,25(OH)_2_D production (17), and low phosphate intake of phosphorus binders caused 35% decrease in plasma FGF23 level (18). However in healthy individuals no changes in FGF23 levels were observed after both phosphate deprivation and loading (19,20).

FGF 23 is also involved in renal sodium handling (21) and, what is even more interesting, it suppresses the expression of angiotensin-converting enzyme-2 (ACE2) in CKD-mice and thereby activates renin-angiotensin-aldosterone system (RAAS) (22). FGF23 can also influence the RAAS indirectly through vitamin D (23), which probably reduces renin gene expression and secretory activity of the juxtaglomerular apparatus, the main place of production of renin (24).

The investigation of the effect of FGF23 on hypertension is not confined to in vitro models. Hypertensive people were found to have significantly higher plasma FGF23 level than normotensive people (25). FGF23 was shown to have an association with markers of inflammation in individuals with CKD stages 2-4 (26), and with impaired endothelium-dependent vasodilatation in healthy individuals and early CKD patients (27). This effect of FGF23 might also result indirectly from a decrease in 1,25 (OH)_2_D (28). FGF23 also correlated with asymmetrical dimethylarginin (ADMA), which is an endogenous inhibitor of NO synthase and a biomarker of endothelial dysfunction (29).

So far, however, the relevance of FGF23 in primary arterial hypertension has been under-investigated. What is more, available data focus on adult hypertensive patients and possible relation of phosphorus intake and increased FGF23 concentration to elevated BP (25). There is a paucity of similar data in children and adolescents. The aims of this research were to determine whether urinary excretion of FGF23 in hypertensive children and adolescents was higher than in healthy controls and whether its urinary level correlated with serum calcium, phosphorus, vitamin D, and PTH concentrations. Reference group data were obtained from the OLAF study, which established the reference blood pressure range for Polish children and adolescents. A strong correlation between serum and urine FGF23 was previously confirmed (r = 0.92, *P* < 0.001) (30).

## Patients and methods

This cohort study included all 42 hypertensive children and adolescents (17 girls and 25 boys) aged 6-18 years who were admitted to the Department of Pediatrics and Nephrology, Medical University of Białystok between January and December 2013. They were referred to our Unit after they had been found to have elevated causal BP by their GPs. The reference group consisted of 46 healthy children and adolescents (17 girls and 29 boys) aged 6-18 years from the OLAF study (n = 40) or children of health professionals from our Unit (n = 6). The data collection in OLAF study was performed between January and November 2009.

Inclusion criteria were age 6-18 years, primary arterial hypertension (confirmed by ambulatory blood pressure monitoring [ABPM]–mean systolic or diastolic daytime or night-time BP higher than or equal to the 95th percentile for age, sex, and height and load systolic or diastolic blood pressure higher than 25%) (31), normal clinical examination, creatinine, and thyroid-stimulating hormone (TSH) levels, no abnormalities in urinalysis, and normal renal ultrasound and electrocardiogram.

Exclusion criteria were heart failure, renal dysfunction, secondary forms of hypertension, diseases of thyroid, parathyroid, or adrenal glands, rickets, systemic inflammatory conditions, autoimmune and hematological diseases, treatment with contraceptive pills, and treatment with vitamin D or calcium. Reference participants did not have a family history of hypertension and other CVDs, renal disease, diabetes, or urolithiasis. The participants’ medical history was taken from their parents.

The study protocol was approved by the Local Committee of Bioethics, Medical University of Bialystok, while the OLAF study was approved by The Children’s Memorial Health Institute Ethics Committee and was performed in accordance with the Declaration of Helsinki.

Body weight and height were measured using a balance beam scale and pediatric wall-mounted stadiometer and body mass index (BMI) was calculated as weight (in kilograms) divided by the square of height (meters squared). Age- and height-specific reference values for BMI were generated by the Lambda-Mu-Sigma (LMS) method (32), with LMS values taken from the OLAF study (33). Waist circumference was measured midway between the top of the hip bone and the bottom of ribs, and hip circumference at the widest portion of the buttocks. The waist-to-hip ratio (WHR) was calculated by dividing waist circumference by hip circumference. BP was measured using an automatic manometer in a sitting position. 3 measurements were obtained in 3-minute intervals and the average of the second and third measurement was calculated. Standard deviation score of systolic and diastolic BP (SDS) was used for statistical analysis. Anthropometric methods have been described thoroughly in OLAF study (34).

In hypertensive participants, serum creatinine, calcium, phosphorus, 25(OH)D, and PTH were measured in blood samples taken after 12 hours of overnight fasting. For the reference group, these values were taken from the OLAF study.

First morning void urine samples were obtained and frozen at-70°C until assaying. Concentration of FGF23 and creatinine was assessed and daily urine excretion of calcium, phosphorus, magnesium, sodium, and creatinine was also estimated. Urine FGF23 concentration was measured using a commercially available enzyme-linked immunosorbent assay (ELISA) kit, Human Intact FGF-23ELISA Kit (Immutopics Inc., San Clemente, CA, USA), according to the manufacturer's instructions. In brief, two polyclonal antibodies specific for FGF23 were used to detect amino-terminal and the carboxyl-terminal portions of FGF23. The antibody detecting NH_2_-terminal parts of FGF23 were conjugated to horseradish peroxidase and followed by color-forming peroxidase substrate containing tetramethylbenzidine. The color was measured at 450 nm by a microtiter plate reader and compared with a standard curve. Urinary FGF23 levels were expressed in relative units per milliliter (RU/mL).

FGF23 measurements were normalized using urine creatinine concentration to account for the influence of urinary dilution. Urine creatinine levels were determined by up-dated Jaffé’s method (35). FGF23 levels were expressed as urine FGF23 to creatinine ratio (FGF23/cr.) in RUs per milligram creatinine (RU/mg cr.). Urine calcium, phosphorus, and magnesium levels were determined with a Cobas-Integra 800 analyzer and Roche reagents (Roche, Indianapolis, IN, USA). Serum creatinine was determined by Jaffe reaction and PTH using Immulite 2000 Intact PTH assay (Siemens, Deerfield, IL, USA). The estimated glomerular filtration rate (eGFR) was calculated from the updated Schwartz formula: eGFR = 0.413 × G (cm)/Lcr (mg/dL), where G is growth and Lcr is serum creatinine level.

ABPM was performed using the oscillometric monitor (Spacelabs Healthcare, Snoqualmie, WA, USA). The monitors were programmed to measure BP every 15 minutes during daytime (8:00-22:00) and every 30 minutes during night-time (22:00-8:00), corrected according to the participants’ diaries if necessary. Recording started between 8 and 9 am and lasted for 24 hours. Recordings with a minimum of 80% of measurement and without breaks longer than 2 hours were used. Mean systolic (sBP) and diastolic blood pressure (dBP) were calculated separately for the 24-h period, awake period, and sleeping period. We also measured load systolic (LSBP) and diastolic blood pressure (LDBP) during the day and night. Hypertension on the basis of ABPM was defined as mean daytime or night-time LSBP or LDBP levels ≥95th percentile and daytime or night-time LSBP or LDBP levels higher than 25%. The values were adjusted by sex and body height according to the reference values (33). Participants or their parents were asked to record the bedtime and waking-up time. After 24 hours, the data downloaded using the manufacturer’s software.

### Statistical methods

Data were analyzed using STATISTICA (StatSoft, Inc., Tulsa, OK, USA), version 10. Categorical variables are expressed as counts (percentage) and continuous variables as median and interquartile range, unless stated otherwise. The Shapiro-Wilk test was used to test the normality of distribution. The groups were compared with χ^2^ test and Fisher exact test for categorical variables and *t* test for continuous variables if normally distributed or Mann-Whitney test if not normally distributed. Correlations between FGF23/cr. and other variables were evaluated by Pearson or Spearman’s test (ρ) as appropriate. *P* < 0.05 was considered statistically significant.

## Results

**Figure 1:**
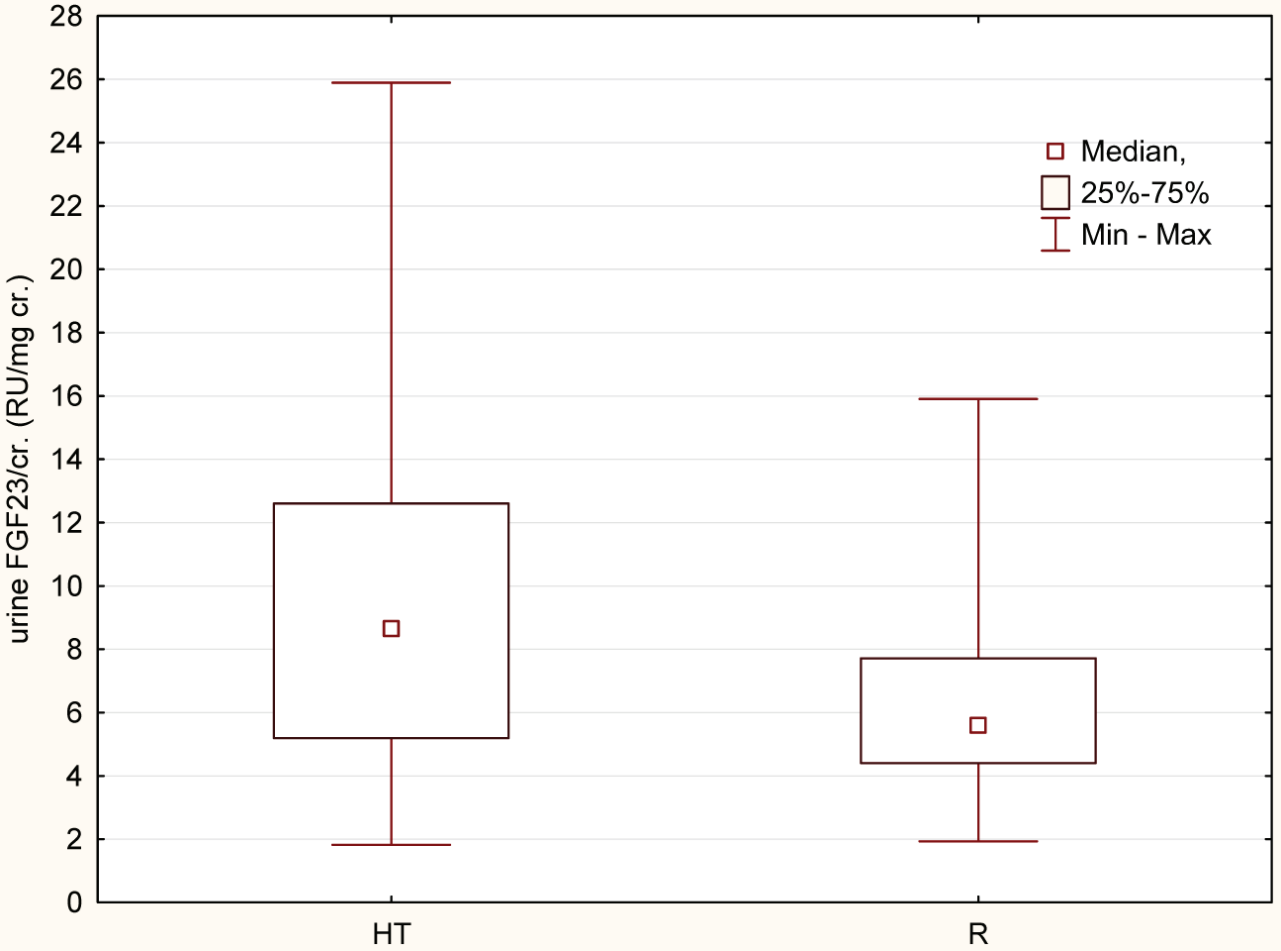
Urine fibroblast growth factor23/creatinine (FGF23/cr) in hypertensive (HT) and reference group (**R**).

Hypertensive participants had significantly higher weight, BMI, standard deviation score of BMI specified by the use of LMS algorithm (BMI SDS), waist and hip circumference, and WHR. They also had significantly higher average of systolic and diastolic BP, sBP SDS and dBP SDS, and urine FGF23/cr. (*P* < 0.001, *P* < 0.001, *P* = 0.007, respectively) (Table 1) (Figure 1).

**Table 1:** Anthropometric, clinical, and metabolic characteristics of hypertensive and reference group*

	Hypertensive (N = 42)	Reference (N = 46)	*P* (Mann-Whitney) test)
Sex (M/F)	25/17	29/17	0.828
Age (years)	17 (16-17)	16 (15-17)	0.730
Height (cm)	163 (143-174)	161.8 (137.1-172)	0.760
Weight (kg)	61.65 (49-80)	52.8 (32-61)	0.005
Body mass index standard deviation score	1.79 (0.74-2.14)	0.19 (-0.21-1.03)	≤0.001
Waist to hip ratio	0.89 (0.81-0.92)	0.82 (0.76-0.84)	≤0.001
Systolic blood pressure standard deviation score	1.5 (0.85-2.17)	0.33 (-0.2-0.72)	≤0.001
Diastolic blood pressure standard deviation score	1.17 (0.82-2.1)	0.43 (0.02-0.9)	≤0.001
Creatinine (mg/dL)	0.58 (0.45-0.74)	-	-
Calcium (mmol/L)	2.55 (2.5-2.6)	-	-
Phosphorus (mg/dL)	4.36 (3.93-5.02)	-	-
25(OH)D (ng/mL)	19 (12-23)	-	-
Parathyroid hormone(pg/mL)	24.6 (18.7-38.5)	-	-
Glomerular filtration raterate(mL/min/L,73m^2^)	111.1 (95.6-131.64)	-	-
Urinary excretion of calcium (mg/kg/24 h)	1.6 (1.13-2.48)	-	-
Urinary excretion of phosphorus (mg/kg/24 h)	11.72 (9.49-14.97)	-	-
Urinary excretion of magnesium (mg/dL)	1.29 (0.95-1.64)	-	-
Urine creatinine (mg/dL)	91.08 (61.98-130.35)	122.64 (87.81-145.49)	0.029
Urine fibroblast growth factor23/creatinine (RU/mg) creatinine)	8.65 (5.19-12.6)	5.59 (4.4-7.71)	0.007

*Values are presented as median and interquartile range.

**Figure 2:**
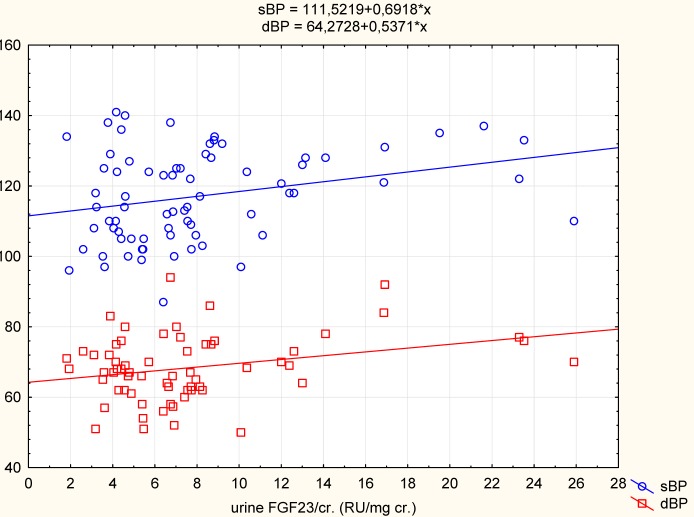
Correlation between FGF23/creatinine and systolic (ρ = 0.281, *P* = 0.022) and diastolic blood pressure (ρ = 0.191, *P* = 0.099) (sBP – blue line, dBP – red line).

We found no significant differences in urine FGF23/cr. excretion between boys and girls. We analyzed the correlations between urine FGF23/cr. and parameters of physical development and BP in all participants and the correlations between FGF23/cr. and biochemical parameters and urine electrolyte excretion in hypertensive participants. Significant positive correlations were found between urine FGF23/cr. and BMI SDS (ρ = 0.231, *P* = 0.035) and sBP SDS (ρ = 0.281, *P* = 0.022) in all participants. However, the correlation between FGF23 and BMI SDS may be a result of higher BMI values in hypertensive children than in the reference group. We observed no significant correlation between FGF23/cr. and dBP or dBP SDS (Figure 2), but we observed a significant positive correlation between FGF23/cr. and serum calcium concentration (ρ = 0.361 and *P* = 0.013).We also observed a significant negative correlation of urine FGF23/cr. with serum 25(OH)D level (ρ = -0.322, *P* = 0.036) and with urinary calcium, phosphorus, and magnesium excretion (respectively, ρ = -0.572, ρ = -0.711, ρ = -0.822; *P* < 0.001) (Table 2).

**Table 2:** Correlations between urine FGF23/creatinine and parameters of physical development, serum concentration of calcium, phosphorus, 25(OH)D, parathyroid hormone, and urinary excretion of calcium, phosphorus and magnesium in hypertensive and reference group

	Correlation coefficient (ρ)	*P*
Age (years)	0.102	0.348
Body mass index standard deviation score(kg/m^2^)	0.231	0.035
Systolic blood pressure standard deviation	0.281	0.022
Diastolic blood pressure standard deviation	0.191	0.099
Serum calcium (mmol/L)	0.361	0.014
Serum phosphorus (mg/dL)	0.154	0.339
Serum 25(OH)D (ng/mL)	-0.322	0.036
Serum parathyroid hormone (pg/mL)	0.034	0.828
Glomerular filtration rate mL/min/1.73m^2^	0.093	0.553
Urinary excretion of calcium (mg/dL)	-0.572	≤0.001
Urinary excretion of phosphorus (mg/dL)	-0.711	≤0.001
Urinary excretion of magnesium (mg/dL)	-0.82	≤0.001

The factors that significantly correlated with urine FGF23/cr. ratio in single regression analyses were included into multiple regression and created two models. In the first one, three parameters (BMI- Z-score, SBP SDS, and urine calcium excretion) accounted for 32% of the variations in FGF23/cr. ratio level (ρ = 0.568, *P* = 0.064) and only calcium excretion was significant (*P* < 0.001). In the second model, four parameters (SBP SDS, serum vitamin D3, and urine calcium and phosphorus excretion) accounted for 30% of the variations in FGF23/cr. ratio level (ρ =  = 0.551, *P* = 0.131) and only calcium excretion was significant (*P* < 0.001).

## Discussion

The most interesting finding of this study was significantly higher concentration of urine FGF23in hypertensive participants than in the reference group and the positive correlation of FGF23 with systolic BP but not diastolic BP. The latter observation may be related to the fact that the majority of children and adolescents in our study had isolated systolic hypertension. Our observation of higher FGF23 levels in hypertensive patients is consistent with previous research on adult population (25). This fact may have some clinical implications. FGF23 might become a target for new antihypertensive drugs, but it needs to be confirmed by further studies, especially on the relation of serum FGF23 and blood pressure regulation.

We found a negative correlation between FGF23 and urinary phosphorus excretion. Other studies found positive correlation between FGF23 and phosphaturia but mainly in CKD patients (13,18), while our study included individuals with a normal GFR. Circulating FGF23 concentration increases with declining renal function in patients with CKD, but does not change in response to variation in phosphate intake in healthy participants. What is more, to assess urine FGF23 concentration, we used an assay detecting a full-length human FGF23, which is better than C-terminal assay that also detects accumulated fragments (36).

Our study found that FGF23 played an important role in the pathogenesis of hypertension in young population. However, we have to take into consideration some study limitations, such as small sample, single-center design, and lack of some biochemical findings like serum creatinine, calcium, phosphorus, PTH, or urinary excretion of calcium phosphorus, magnesium in the reference group. Therefore, our results have to be confirmed by further research in the following areas: 1. measurement of serum FGF23 in hypertensive children and adolescents; 2. assessment of the possible relationship between FGF23 and activation of RAAS; and 3. assessment of the correlation between FGF23 and reactive oxygen species production and nitric oxidesynthesis.
